# Establishment of an Efficient Genome Editing System in Lettuce Without Sacrificing Specificity

**DOI:** 10.3389/fpls.2022.930592

**Published:** 2022-06-22

**Authors:** Wenbo Pan, Xue Liu, Dayong Li, Huawei Zhang

**Affiliations:** ^1^Peking University Institute of Advanced Agricultural Science, Weifang, China; ^2^School of Advanced Agricultural Sciences, Peking University, Beijing, China; ^3^National Engineering Research Center for Vegetables, Beijing Vegetable Research Center, Beijing Academy of Agriculture and Forestry Science, Beijing, China; ^4^Beijing Key Laboratory of Vegetable Germplasm Improvement, Beijing, China

**Keywords:** genome editing, CRISPR/Cas9, intron-mediated enhancement, *GRF5*, lettuce

## Abstract

The efficiency of the CRISPR/Cas9 genome editing system remains limited in many crops. Utilizing strong promoters to boost the expression level of *Cas9* are commonly used to improve the editing efficiency. However, these strategies also increase the risk of off-target mutation. Here, we developed a new strategy to utilize intron-mediated enhancement (IME)-assisted *35S* promoter to drive *Cas9* and sgRNA in a single transcript, which escalates the editing efficiency by moderately enhancing the expression of both *Cas9* and sgRNA. In addition, we developed another strategy to enrich cells highly expressing *Cas9*/sgRNA by co-expressing the developmental regulator gene *GRF5*, which has been proved to ameliorate the transformation efficiency, and the transgenic plants from these cells also exhibited enhanced editing efficiency. This system elevated the genome editing efficiency from 14–28% to 54–81% on three targets tested in lettuce (*Lactuca sativa*) without increasing the off-target editing efficiency. Thus, we established a new genome editing system with highly improved on-target editing efficiency and without obvious increasement in off-target effects, which can be used to characterize genes of interest in lettuce and other crops.

## Introduction

The CRISPR/Cas9 system is a powerful genome editing tool that has been widely used in the past decade (Gao, 2021). With the complementary base pairing mechanism, the Cas9 endonuclease is guided to the specific DNA sequence by the guide RNA (gRNA), and generates double-stranded DNA breaks (DSBs) at the desired loci. Predominantly, the DSBs are repaired by the error-prone non-homologous end joining (NHEJ) pathway, which introduces insertions/deletions (indels) that range from one to hundreds of base pairs, that could lead to site-specific genetic alterations (Gao, 2021; Hassan et al., 2021). Until today, this technology has been successfully used to generate mutant plants and for agronomic trait enhancement in many crops. Nevertheless, the editing efficiency remains quite limited in several vegetable and crop plants.

Boosting the expression of *Cas9* or sgRNA is the major method to improve the genome editing efficiency (Castel et al., 2019; Hassan et al., 2021). Several studies have utilized strong promoters, such as the *RPS5A* promoter (Tsutsui and Higashiyama, 2017; Castel et al., 2019; Ordon et al., 2020; Oh and Kim, 2021), the *UBQ10* promoter (Wang and Chen, 2019; Wolabu et al., 2020), and the *MAS* promoter (An et al., 2021), to strengthen the expression level of *Cas9*, which leads to increasements in the genome editing efficiency. Also, the augmentation of sgRNA level, by using native U6/U3 promoters (Sun et al., 2015; Ren et al., 2021), or by using Pol II promoters such as the *ubiquitin* promoter (Ding et al., 2018), or the *cestrum yellow leaf curling virus* (*CmYLCV*) promoter (Cermak et al., 2017; Li et al., 2021), escalates genome editing efficiency. Also, the *Cas9* with multiple introns efficiently generates more mutants than the conventional *Cas9* (Grutzner et al., 2021). However, these strategies also increase the risk of off-target mutation, which might interfere phenotypic analysis of desired genes and more severely hinder deregulation and commercial release of genome-edited crops.

Intron-mediated enhancement (IME) is a well-known phenomenon to enhance homogeneous protein expression in plants and animals (Vain et al., 1996; Laxa, 2016). The introns located in the 5′-UTR region from several strong and constitutive genes, such the first intron of *UBQ10, ACTIN, TRP1* (Rose, 2004; Jeong et al., 2009), have been proved to greatly improve the expression of downstream gene. For example, the first intron of maize *ubiquitin 1* (*ZmUbi1*) located in the 5′-UTR region combines with CaMV *35S* promoter leads to a over 90-fold increasement of gene expression in maize and bluegrass (Vain et al., 1996). Many works have attempted to identify the key cis-elements in this process, but the detailed mechanism is still not clear, since it has been found that the sequence and splicing process are not the key feathers of these introns (Rose and Beliakoff, 2000; Rose, 2004; Back and Walther, 2021). Thus, it’s promising to engineer these introns to enhance the strength of the promoters that drive the expression of the CRISPR/Cas9 system.

Several *DR* (*DEVELPMENTAL REGULATOR*) genes, such as the *WUS* (*WUSCHEL*), *BBM* (*BABY BOOM*) and *GRFs* (*GROWTH-REGULATING FACTORs*), have been proved to improve the transformation efficiency (Lowe et al., 2016; Debernardi et al., 2020; Kong et al., 2020; Qiu et al., 2022). Ectopic expression of the *BBM* gene, which is originally identified in *Brassica napus*, has diverse functions in plant cell proliferation, growth and development (Jha and Kumar, 2018). The co-expression of *BBM* with *WUS* greatly boosts the transformation efficiency of several monocot species, including rice, maize and sorghum (Lowe et al., 2016). Several plant-specific GRF transcription factors have successfully elevated the regeneration and transformation efficiency of crop plants, such as soybean, canola, and sunflower (Kong et al., 2020; Pan et al., 2022). The overexpression of a chimeric protein consisting of the GRF4 and GRF-interacting factor 1 (GIF1) proteins reinforce the regeneration efficiency and regeneration speed in wheat, triticale, rice and watermelon (Debernardi et al., 2020; Feng et al., 2021; Qiu et al., 2022). However, the effect of these DRs on the genome editing efficiency in the regenerated plants has not been investigated.

Lettuce is one of the most popular vegetable crops that is cultivated worldwide (Su et al., 2020; Assefa et al., 2021). The substantial amounts of ascorbic acid, vitamin A, carotenoids, folate, and other primary and secondary metabolites are beneficial to human health (Assefa et al., 2021). However, the candidate genes behinds these traits are poorly investigated. The CRISPR/Cas9 genome editing system, which is powerful and promising in generating the desired mutants and in crop breeding, has been utilized in the studies of lettuce in recent years (Bertier et al., 2018; Zhang et al., 2018; Luo et al., 2021). However, an improved and efficient genome editing has not been established for lettuce and is urgently needed.

In this work, we established an intron-mediated enhancement-based strategy to increase the expression of *Cas9* and sgRNA, and also tested the effect of *GRF5* on lettuce (*Lactuca sativa*) transformation and genome editing. These two methods successfully boosted the genome editing efficiency from 14–28% to 54–81% on three targets tested in lettuce without increasing the off-target editing efficiency.

## Results

### IME-Mediated Enhancement of Cas9 and sgRNA Expression

**FIGURE 1:**
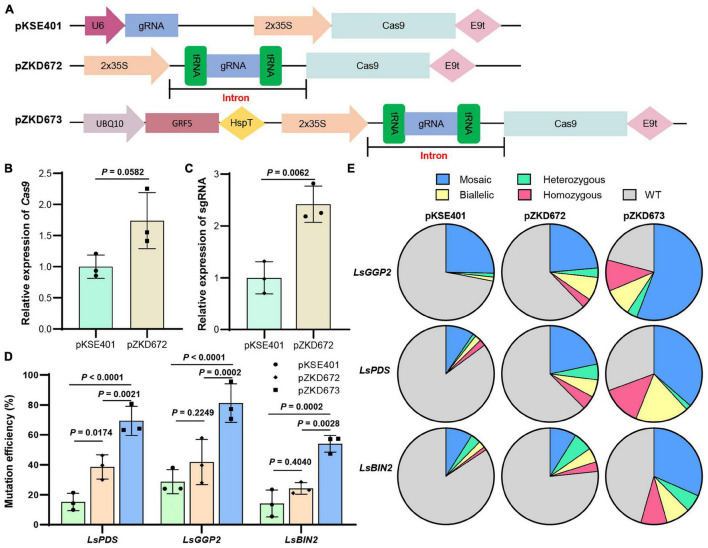
Boost genome editing efficiency through intron-mediated enhancement and *GRF5* co-expression. **(A)** The schematic diagram of the vectors. In the conventional genome vector pKSE401, sgRNA is driven by the *U6* promoter, and *Cas9* is driven by the CaMV *35S* promoter. In pZKD672, engineered intron contain sgRNA was used to boost *Cas9* and sgRNA expression through the intron-mediated enhancement mechanisms. The endogenous tRNA-processing system was used to generate mature sgRNA. In pZKD673, the *GRF5* driven by the *UBQ10* promoter was added to the genome editing vector to facilitate the screening of plants with high expression levels. **(B,C)** The expression level of *Cas9* **(B)** and sgRNA **(C)** in lettuce protoplasts using the pKSE401 or the pZKD672 vector. *LsACT* was used as the internal control. The *P* value was calculated with paired two-tailed Student’s *t*-test. **(D)** The mutation ratio of three tested target sites using the indicated vectors in transgenic lettuce plants in the T0 generation. The *P* value was calculated with Two-way ANOVA test. **(E)** The proportion of different mutation types among all the transgenic plants from three replicates.

First, we decided to moderately enhance the expression of both *Cas9* and sgRNA through IME. Previous studies have successfully engineered the sgRNA expression cassette tRNA-sgRNA-tRNA into the first intron of *ZmUbi1*, thus the mature sgRNA can be generated by the endogenous tRNA-processing system (Xie et al., 2015; Zhong et al., 2020). We generated pZKD672 by inserting this engineered intron (Supplementary Table 1) into a *35S* promoter-driven Cas9 expression cassette (Figure 1A). In this way, Cas9 and sgRNA were driven by an IME-assisted *35S* promoter and co-expressed from a single transcriptional unit. We speculated that the expression levels of both *Cas9* and sgRNA could be improved. The pKSE401 vector, in which *Cas9* and sgRNA are respectively driven by *35S* promoter and *Arabidopsis U6-26* promoter (Xing et al., 2014), was used as the control.

To test our hypothesis, we utilized pKSE401 and pZDK672 to construct an sgRNA targeting *LsPDS* (*PHYTOENE DESATURASE*) in lettuce, and examined the expression of *Cas9* and sgRNA in lettuce protoplasts. Compared with those from pKSE401, transcript levels of *Cas9* and sgRNA from pZDK672 were increased by 0.74- and 1.42-fold, respectively (Figures 1B,C), proving the power of IME in moderately enhancing the expression of Cas9 and sgRNA.

### IME Boosts the Genome Editing Efficiency in Transgenic Lettuce Plants

To investigate whether the pZDK672 could elevate the editing efficiency in stable transgenic plants, we selected *LsPDS* and two additional target genes, *LsBIN2* (*BR-INSENSITIVE 2*) and *LsGGP2* (*GDP-L-GALACTOSE PHOSPHORYLASE*) to generate stable transgenic lettuces through *Agrobacterium*-mediated transformation (Zhang et al., 2018). The average mutation efficiencies by the pZKD672 were 38.59, 41.85, and 24.23% for *LsPDS*, *LsGGP2*, and *LsBIN2* in 3 biological repeats, respectively, while only 15.24, 28.74, and 14.20% transgenic plants were mutated by pKSE401 (Figure 1D and Supplementary Tables 2, 3), with 1.53-, 0.80-, and 0.71-fold increasement. It suggests that the moderate magnification in the expression of *Cas9* and sgRNA through IME resulted in a weak augmentation in editing efficiency.

### Optimizing the Genome Editing Efficiency by the *GRF5* Co-expression

Next, we hoped to optimize pZKD672 to further elevating its editing efficiency. The plant genetic transformation often generates populations with diverse gene expression levels. In the plant genome editing processes, the cells with higher expression levels of *Cas9* and sgRNA, which leads to higher mutation rates, are the desired ones for regeneration. Direct enrichment of these cells or plants could also increase the mutation efficiency, such as the *GLABRA2* mutation-based visible selection (GBVS) system which adds the *GL2* target as a visible selection marker to identify plants with high mutation efficiency (Kong et al., 2021). However, mutation of a second gene might be a concern for crop breeding. This prompted us to explore novel strategies to enrich these cells. Several *DRs* have been proved to improve the transformation efficiency by promoting the somatic embryogenesis or regeneration rates (Lowe et al., 2016; Kong et al., 2020; Qiu et al., 2022). They have been widely used in plants recalcitrant to transformation (Kong et al., 2020; Pan et al., 2022; Qiu et al., 2022). However, the effect of these *DRs* on the editing efficiency of recipient plants during stable transformation had not yet been investigated. We surmised that the expression level of *DRs* should be correlated with that of *Cas9* and sgRNA when they were constructed in a single T-DNA. The cells, highly expressing *DRs*, *Cas9* and sgRNA, could gain an advantage over other cells to redifferentiation, hence co-expression of DRs could elevate mutation rate during stable transformation. To verify our speculation, we added an *Arabidopsis GRF5* overexpression cassata (Supplementary Table 1) to pZKD672 to generate pZKD673 (Figure 1A). pZKD673 with corresponding target spacers were also transformed into lettuce. The mutation efficiencies of *LsPDS, LsGGP2*, and *LsBIN2* by pZKD673 are 69.38, 81.22, and 54.00% in three biological replicates, respectively, and exhibit 0.80-, 0.94-, and 1.23-fold increase, compared with 38.59, 41.85, and 24.23% by pZKD672, respectively (Figure 1D and Supplementary Tables 2, 3). To further prove our postulate, we randomly selected about 24 transgenic plants for each vector and mixed them into 3 samples to check the expression level of *Cas9*. The result showed that the expression of Cas9 in pZKD673 transgenic plants is about 2.58-fold higher than the pZKD672 vector (Supplementary Figure 1). Our data indicate that co-expressing *GRF5* could improve the editing efficiency of pZKD672.

Among all the T0 transgenic plants, the ratio of null mutants (homozygous and biallelic) was also increased. For example, the amount of *lspds* null mutants was raised from 4.23% for pKSE401 to 10.53% for pZKD672 and 31.21% for pZKD673 (Figure 1E and Supplementary Table 3). These results demonstrated that our new vectors could generate more null mutants, which are suitable for phenotyping or breeding in the offspring, and are labor- and time-saving.

### Off-Target Analysis of the New Genome Editing Vectors

Finally, to evaluate off-targeting efficiency, five predicted highly risky off-target sites for each target gene were identified through the CRISPOR program^1^ (Haeussler et al., 2016), and 20 on-target mutant lines for each plasmid were examined (except for the *LsBIN2* by the pKSE401, with only 14 mutants obtained). No off-target mutation was detected, even at the off-target 1 (OT1) and OT2 of *LsGGP2*, and the OT1 of *LsBIN2*, which have 2 mismatches with the corresponding target sequence (Table 1 and Supplementary Table 4). These results indicated our newly established systems do not increase off-target efficiency.

**TABLE 1:** The off-target analysis results.

Target	Off-Target sites	Sequence (5′–3′)	No. mismatch	CFD Score*^a^*	No. off-target mutant from 20 on-target mutant plants*^b^*		

					pKSE401	pZKD672	pZKD673
*LsPDS*	On-target	GGCCACCGAGTGACTCGATGTGG	0	1			
	OT1	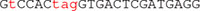	4	0.34	0/20	0/20	0/20
	OT2	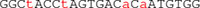	4	0.27	0/20	0/20	0/20
	OT3	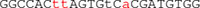	4	0.08	0/20	0/20	0/20
	OT4	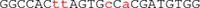	4	0.06	0/20	0/20	0/20
	OT5	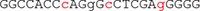	4	0.01	0/20	0/20	0/20
*LsGPP2*	On-target	ACGACAAGTTGCAGACATCACGG	0	1			
	OT1	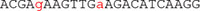	2	0.42	0/20	0/20	0/20
	OT2	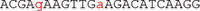	2	0.42	0/20	0/20	0/20
	OT3	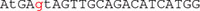	3	0.39	0/20	0/20	0/20
	OT4	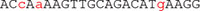	3	0.02	0/20	0/20	0/20
	OT5	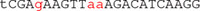	4	0.42	0/20	0/20	0/20
*LsBIN2*	On-target	ATCACAGTGATGCTCGTCAAAGG	0	1			
	OT1	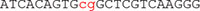	2	0.4	0/14	0/20	0/20
	OT2	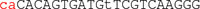	3	0.5	0/14	0/20	0/20
	OT3	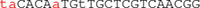	4	0.74	0/14	0/20	0/20
	OT4	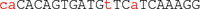	4	0.5	0/14	0/20	0/20
	OT5	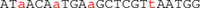	4	0.41	0/14	0/20	0/20

*^a^The CFD score indicates the potential of off-target editing (Haeussler et al., 2016). ^b^Only 14 mutants were obtained by the pKSE401 vector at the LsBIN2 site.*

## Discussion

In this study, we established a new genome editing system for creating mutations with high frequency in lettuce. With an intron expressing the sgRNA, and GRF5-mediated enrichment, we dramatically boosted the mutation efficiency compared with the commonly used vector in transgenic lettuce plants.

Successful engineering of introns to express sgRNA has been reported in other studies (Ding et al., 2018; Zhong et al., 2020). In these studies, the intron is inserted into the 5′-UTR or within the coding region of *Cas9*. All these experiments were conducted in rice, and compared to traditional genome editing vectors, these sgRNA containing introns didn’t significantly improved the editing efficiency (Ding et al., 2018; Zhong et al., 2020). This is probably because that the rice *UBQ10* promoter or the maize *Ubi* promoter is used to drive Cas9. These Ubiquitin promoters itself contains the introns with IME. Therefore, additional adding of another IME introns probably doesn’t make functions. In this work, the genome editing is conducted in a dicot plant lettuce, and the most widely used *35S* promoter is used to drive Cas9. It has been well proved that adding an IME intron could significantly boost the power of *35S* promoter (Vain et al., 1996; Laxa, 2016). Our result proved that this modified intron could indeed improve the activity of the *35S* promoter that expresses the *Cas9*. And it should be pointed out that the IME intron strategy might not be applicable to all the genome editing vectors, and not all the plant species.

In order to generate mature sgRNAs within the intron, the tRNA sequence was placed upstream and downstream of the spacer-sgRNA sequence. It has been well proved that these polycistronic gene can be processed by the endogenous tRNA-processing enzyme RNAse Z and RNAse P (Xie et al., 2015; Zhong et al., 2020). Successfully genome editing of endogenous targets in our experiments also confirmed these results. In addition to the tRNA-processing system, other sgRNA processing system, such as the dual HH-HDV ribozyme system (Gao and Zhao, 2014), or the sequence-specific RNase Csy4 (Przybilski et al., 2011), has also been used to express sgRNA in plants. In these experiments, the tRNA-processing system enables efficient sgRNA expression by the Pol II promoters, and efficient multiplex genome editing (Xie et al., 2015; Li et al., 2021). Also, the tRNA-processing system exhibited higher or comparable processing efficiency and mutation rates than the ribozyme system and the Csy4 system in these experiments (Tang et al., 2019; Hsieh-Feng and Yang, 2020; Zhong et al., 2020). Thus, our new vectors are promising in efficient multiplex genome editing in lettuce, and other dicot plants.

The power of DRs in genetic transformation has been observed in many plant species. With the assistance of DRs such as *GRF5*, efficient and genotype-independent transformation can be achieved without obvious growth abnormities, and this system has been used to generate mutants by the CRISPR/Cas9 genome editing system (Debernardi et al., 2020; Pan et al., 2022). In our previous experiments, we demonstrated that *GRF5* outperforms other DR genes, such as *GRF4-GIF1*, *BBM* and *WUS*, in the genetic transformation of watermelon (*Citrullus lanatus*) (Pan et al., 2022). However, the effect of GRF5 on the genome editing efficiency has not been investigated. In our work, we proved that overexpressing the *GRF5* gene could dramatically elevate the genome editing efficiency. We suspect that this is a transgenic enrichment effect: as the GRF5 and Cas9 are constructed in a single T-DNA, efficient expression of Cas9 should co-relate with efficient expression of DRs, which facilitates the regeneration process. Thus, most of the transgenic plants we obtained with the pZKD673 vectors should have higher mutation efficiency. Our observation that the expression level of *Cas9* is indeed higher in pZKD673 transgenic plants than the pZKD672 transgenic plants confirmed our hypothesis. Very recently, another group also observed the same mutation efficiency increasement by co-expressing *WUS* in sorghum (Che et al., 2022), but the detailed mechanism hasn’t been revealed. These works revealed the power of DRs, not only on genotype-independent genetic transformation, but also in efficient genome editing.

With great improvement in the genome editing efficiency, we can easily obtain large number of mutants. And the efficiency amplification also leads to higher ratio of homozygous and biallelic mutants in the T0 generation. What’s more, we didn’t observe significant increasement in the off-target mutation efficiency. Thus, these homozygous and biallelic mutants could directly be used for phenotype analysis and functional verification, which could save plenty of time and efforts.

In summary, we utilized two novel strategies, IME-mediated the moderate enhancement of *Cas9*/sgRNA expression and *DR* gene-associated transgene enrichment, to establish a highly efficient plant genome editing system without obvious off-targeting increase. These strategies could also be applied in other genome editing tools, such as base-editors and the prime-editors, and other crop species, to boost the editing efficiency.

## Materials and Methods

### Plant Materials

The *L. sativa* L. var. capitata 101 was bought from Jingyan Yinong (Beijing) Seed Sci-Tech Co., Ltd. Plants were grown under a photoperiod of 16 h light (150 μmol m^–2^ s^–1^) and 8 h dark at 25°C.

### Vector Construction

The PTG sequence and codon optimized GRF5 coding sequence were synthesized at Sangon Biotech. The conventional CRISPR/Cas9 vector pKSE401 (Xing et al., 2014) were used as the control. The pKSE401 vector was first digested by *Hin*dIII, and the 14.5 kb backbone were ligated by T4 DNA ligase. The product was then digested by *Xba*I, and Gibson assembled (Sangon Biotech) with the PTG product amplified with the primer pair PTG-F/PTG-R, generating the pZKD672 vector.

The Arabidopsis UBQ10 promoter, the Arabidopsis Hsp terminator and codon optimized GRF5 coding sequence were amplified with UBQ10p-F/UBQ10p-R, HspT-F/HspT-R and GRF5-F/GRF5-R, respectively. The PCR products were then used as the template and amplified with UBQ10p-F/HspT-R. The 2.75 kb produce were then Gibson assembled with *Hin*dIII digested pZKD672, generating the pZKD673 vector.

The pKSE401, pZKD672, and pZKD673 vector were digested with *Bsa*I, and ligated with annealed target oligos.

The primers were listed in Supplementary Table 5.

### Protoplast Transfection and Analysis

The lettuce protoplast preparation and transfection were performed according to a previous established method (Woo et al., 2015) with some modifications. Briefly, the heart of *L. sativa* L. var. capitata L 101 was sliced with double sides razor blades. The leaves were then digested with 1% Cellulase R10, 0.25% Macerozyme R10, 0.4 M Mannitol, 20 mM KCl, 20 mM MES pH 5.7, 20mM KCl for about 4 h. the enzyme solution was filtered with Miracloth (CALBIOCHEM), and collected by Centrifuged for 1 min at 100 *g*. The protoplasts were washed twice by 10 mL W5 solution (154 mM NaCl, 125 mM CaCl_2_, 5 mM KCl, 2 mM MES pH 5.7. Then the protoplasts were suspended by 10 mL W5 solution and stand on ice for 30 min. The supernatant was removed and the MMG solution (0.4 M Mannitol, 15 mM MgCl_2_, 4 mM MES pH 5.7) was added to a final concentration of 2 × 10^4^ to 2 × 10^5^ cell/ml.

Ten microgram vector were mixed with 200 μL protoplast and mixed gently. Then 220 μL PEG/CaCl_2_ solution (40% PEG4000, 0.2 M Mannitol, 100 mM CaCl_2_) was added and mixed gently. The transfection was performed at room temperature for 15 min and stopped by adding 1.5 mL W5 solution and mixed gently. The protoplast was then washed by 1 mL W5 solution and risen in 200 μL. Then the protoplasts were kept at dark for 2 days at 24°C.

The protoplasts were collected and total RNA were extracted with the Ultrapure RNA Kit (CWbio). The RNA was reverse transcript with the FastQuant RT Kit (With gDNase) (Tiangen) with some modification: 0.5 μL 10 μM qRT-LsPDS-R primer was added to a final 20 μL reverse transcription mixture. The quantitative real-time PCR were performed with CFX Opus real-time PCR system (BioRad) with the Talent qPCR PreMix (SYBR Green) (Tiangen). The corresponding primers were listed in supplementary Table 5.

### Lettuce Transfection

The protocol for lettuce transfection was previously described (Zhang et al., 2018). In brief, surface sterilized lettuce seeds were placed on MS medium and incubated under a photoperiod of 16 h light (150 μmol m^–2^ s^–1^) and 8 h dark at 25°C. The cotyledons were excised from germinated seedlings and incubated for 10 min with the *Agrobacterium* (EHA105) suspension carrying the desired construct. The treated explants were placed on MS co-cultivation medium (MS supplemented with 30 g L^–1^ sucrose, 0.8% plant agar, 0.1 mg L^–1^ α-naphthalaneacetic acid, and 0.5 mg L^–1^ 6-benzylaminopurine) and incubated at 25°C in dark for 48 h.

Afterward, explants were transferred to MS selection medium (MS supplemented with 30 g L^–1^ sucrose, 0.8% plant agar, 0.1 mg L^–1^ α-naphthalaneacetic acid, 0.5 mg L^–1^ 6-benzylaminopurine, 40 mg/l kanamycin monosulfate, and 250 mg L^–1^ carbenicilin), and incubated under a 16 h light/8 h dark cycle at 25°C. After about 25 days, regenerated shoots were excised and transferred to MS rooting medium (1/2 MS supplemented with 15 g L^–1^ sucrose, 20 mg L^–1^ kanamycin monosulfate, and 250 mg L^–1^ carbenicilin) for root induction. The plantlets with well-developed shoot and root were transferred to soil and further examined.

### Analysis of the Genome Editing Efficiency and Potential Off-Target Edits

The genomic DNA of regenerated lettuce plants was extracted with the CTAB method. Positive transgenic plants were examined with the Cas9-check-F2/Cas9-Check-R2 primer pair. The target regions were amplified with corresponding primer pairs, and the analyzed with Sanger sequencing. The sequencing chromatogram were decoded with the TIDE program^2^ (Brinkman et al., 2014).

The potential off-target editing sites were chosen through the CFD score in the CRISPOR program^3^(Haeussler et al., 2016). For each vector, 20 mutant plants were randomly chosen and the target regions were amplified with the corresponding primer pairs. The PCR products were analyzed with Sanger sequencing.

The primers were listed in Supplementary Table 5.

## Data Availability Statement

The original contributions presented in this study are included in the article/Supplementary Material, further inquiries can be directed to the corresponding authors.

## Author Contributions

HZ conceived the study and agreed to serve as the author responsible for contact and ensures communication. HZ and DL supervised the research. WP and XL performed all experiments and analyzed the data with help from HZ and DL. All authors contributed to the article and approved the submitted version.

## Conflict of Interest

The authors declare that the research was conducted in the absence of any commercial or financial relationships that could be construed as a potential conflict of interest.

## Publisher’s Note

All claims expressed in this article are solely those of the authors and do not necessarily represent those of their affiliated organizations, or those of the publisher, the editors and the reviewers. Any product that may be evaluated in this article, or claim that may be made by its manufacturer, is not guaranteed or endorsed by the publisher.

## Footnotes

1
http://crispor.tefor.net/crispor.py

2
http://shinyapps.datacurators.nl/tide/

3
http://crispor.tefor.net/crispor.py
