# Supplementary material for: Establishment of an Efficient Genome Editing System in Lettuce Without Sacrificing Specificity
Source: Front Plant Sci. 2022 Jun 22;13:930592. doi: 10.3389/fpls.2022.930592 (PMC9257259; doi:10.3389/fpls.2022.930592)
Supplement: Supplementary file 1 [file Data_Sheet_1.PDF]

Supplementary Data for

Establishment of an efficient genome editing system in lettuce without sacrificing specificity

Wenbo Pan[1,3†], Xue Liu [2,4†], Dayong Li [2,4]* and Huawei Zhang[1]*

[1] Peking University Institute of Advanced Agricultural Science, 261325, Weifang, China

2 National Engineering Research Center for Vegetables, Beijing Vegetable Research Center, Beijing Academy of Agriculture and Forestry Science, Beijing, 100097, China

[3] School of Advanced Agricultural Sciences, Peking University, Beijing, 100871, China

[4] Beijing Key Laboratory of Vegetable Germplasm Improvement, Beijing 100097, China

[†]These authors contributed equally to this work.

*Correspondences: Dayong Li (lidayong@nercv.org); Huawei Zhang (huawei.zhang@pku-iaas.edu.cn, Dr. Zhang is fully responsible for the distribution of all materials associated with this article)

Contents:

Supplemental Figure 1. The expression level of *Cas9* in transgenic plants harboring the pZKD672 and pZKD673 vector.

Supplemental table 1. The sequences used in this study.

Supplemental table 2. Details of the mutation analysis result.

Supplemental table 3. Summary of the mutation types by each vector.

Supplemental table 4. Details of the predicted off-target sites.

Supplemental table 5. The primers used in this study.

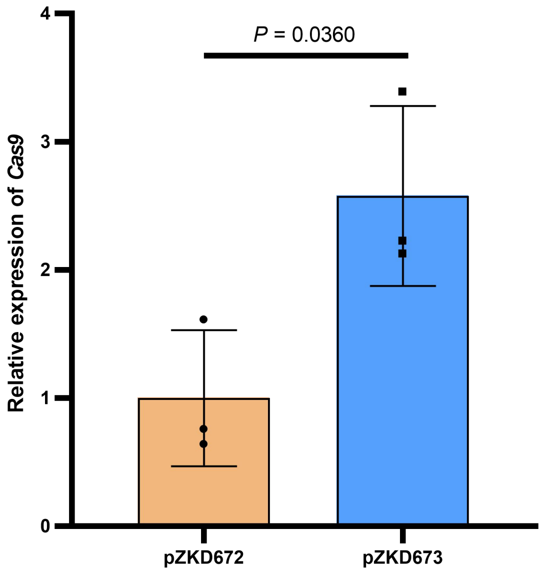

Supplemental Figure 1. The expression level of *Cas9* in transgenic plants harboring the pZKD672 and pZKD673 vector.

For each vector, about 8 transgenic plants were randomly selected and mixed as one sample. Three samples were used to check the expression level of Cas9 by quantitative Real-time PCR (qRT-PCR). The expression level of Cas9 from pZKD672 vectors was normalized to 1. *LsACT* was used as the internal control. The *P* value was calculated with paired two-tailed Student's *t* test.

Supplemental table 1. The sequences used in this study.

>The 2*35S-PTG sequence used in this study. (The HindIII and XbaI sites are marked in cyan. The 2*35S sequence is marked in purple, the tRNA sequence is marked in green, and the sgRNA sequence is marked in brown.)
aagcttgcatgcctgcaggtcaacatggtggagcacgacacacttgtctactccaaaaatatcaaagatacagtctcagaa
gaccaaagggcaattgagacttttcaacaaagggtaatatccggaaacctcctcggattccattgcccagctatctgtcacttt
attgtgaagatagtggaaaaggaaggtggctcctacaaatgccatcattgcgataaaggaaaggccatcgttgaagatgc
ctctgccgacagtggtcccaaagatggacccccacccacgaggagcatcgtggaaaaagaagacgttccaaccacgtct
tcaaagcaagtggattgatgtgataacatggtggagcacgacacacttgtctactccaaaaatatcaaagatacagtctcag
aagaccaaagggcaattgagacttttcaacaaagggtaatatccggaaacctcctcggattccattgcccagctatctgtca
ctttattgtgaagatagtggaaaaggaaggtggctcctacaaatgccatcattgcgataaaggaaaggccatcgttgaagat
gcctctgccgacagtggtcccaaagatggacccccacccacgaggagcatcgtggaaaaagaagacgttccaaccacg
tcttcaaagcaagtggattgatgtgatatctccactgacgtaagggatgacgcacaatcccactatccttcgcaagacccttcc
tctatataaggaagttcatttcatttggagaggacctcgacctcaacacaacatatacaaaacaaacgaatctcaagcaatc
aagcattctacttctattgcagcaatttaaatcatttcttttaaagcaaaagcaattttctgaaattttcaccatttacgaacgata
ctcgagtaaatcagctccacccccgaaaaatttctccccaatctcgcgaggctctcgtcgtcgaatcgaatcctctcgcgtcct
caaggtacgctgcttctcctctcctcgcttcgtttcgattcgatttcggacggcaaacaaagcaccagtggtctagtggtagaat
agtaccctgccacggtacagacccgggttcgattcccggctggtgcatgagacctgaattcaggtctcaGTTTTAGAG
CTAGAAATAGCAAGTTAAAATAAGGCTAGTCCGTTATCAACTTGAAAAAGTGGCACCG
AGTCGGTGCaacaaagcaccagtggtctagtggtagaatagtaccctgccacggtacagacccgggttcgattccc
ggctggtgcagtttgctgtttgatccgttgttgtgtccttaatcttgtgctagttcttaccctatctgtttggtgattatttcttgcagattct
aga

> *GRF5* overexpression cassata (The Arabidopsis *UBQ10* promoter is marked in blue, the codon optimized GRF5 CDS is in black, and the Arabidopsis Hsp terminator is in green)
GATCAGGATATTCTTGTTTAAGATGTTGAACTCTATGGAGGTTTGTATGAAC
TGATGATCTAGGACCGGATAAGTTCCCTTCTTCATAGCGAACTTATTCAAA
GAATGTTTTGTGTATCATTCTTGTTACATTGTTATTAATGAAAAAATATTATTG
GTCATTGGACTGAACACGAGTGTTAAATATGGACCAGGCCCCAAATAAGA
TCCATTGATATATGAATTAAATAACAAGAATAAATCGAGTCACCAAACCACT
TGCCTTTTTTAACGAGACTTGTTCACCAACTTGATACAAAAGTCATTATCCT
ATGCAAATCAATAATCATACAAAAATATCCAATAACACTAAAAAATTAAAAGA
AATGGATAATTTCACAATATGTTATACGATAAAGAAGTTACTTTTCCAAGAAA
TTCACTGATTTTATAAGCCCACTTGCATTAGATAAATGGCAAAAAAAAACAA
AAAGGAAAAGAAATAAAGCACGAAGAATTCTAGAAAATACGAAATACGCTT
CAATGCAGTGGGACCCACGGTTCAATTATTGCCAATTTTCAGCTCCACCG
TATATTTAAAAAATAAAACGATAATGCTAAAAAAATATAAATCGTAACGATCG
TTAAATCTCAACGGCTGGATCTTATGACGACCGTTAGAAATTGTGGTTGTC
GACGAGTCAGTAATAAACGGCGTCAAAGTGGTTGCAGCCGGCACACACG
AGTCGTGTTTATCAACTCAAAGCACAAATACTTTTCCTCAACCTAAAAATAA
GGCAATTAGCCAAAAACAACTTTGCGTGTAAACAACGCTCAATACACGTG
TCATTTTATTATTAGCTATTGCTTCACCGCCTTAGCTTTCTCGTGACCTAGT
CGTCCTCGTCTTTTCTTCTTCTTCTTCTATAAAACAATACCCAAAGAGCTCT

TCTTCTTCACAATTCAGATTTCAATTTCTCAAAATCTTAAAAACTTTCTCTCA
ATTCTCTCTACCGTGATCAAGGTAAATTTCTGTGTTCCTTATTCTCTCAAAA
TCTTCGATTTTGTTTTCGTTCGATCCCAATTTCGTATATGTTCTTTGGTTTA
GATTCTGTTAATCTTAGATCGAAGACGATTTTCTGGGTTTGATCGTTAGATA
TCATCTTAATTCTCGATTAGGGTTTCATAGATATCATCCGATTTGTTCAAATA
ATTTGAGTTTTGTCGAATAATTACTCTTCGATTTGTGATTTCTATCTAGATCT
GGTGTTAGTTTCTAGTTTGTGCGATCGAATTTGTCGATTAATCTGAGTTTTT
CTGATTAACAGATGATGAGTCTAAGTGGAAGTAGCGGGAGAACAATAGGA
AGGCCTCCATTTACACCAACACAATGGGAAGAACTGGAACATCAAGCTCT
AATCTACAAGTACATGGTTTCTGGTGTTCCTGTCCCACCTGAACTCATCTT
CTCCATTAGAAGATCATTGGACACTTCCTTGGTTTCTAGGCTCCTTCCTCA
CCAATCCCTTGGATGGGGGTGTTACCAGATGGGATTTGGGAGAAAACCA
GATCCAGAGCCAGGAAGATGCAGAAGAACAGATGGTAAGAAATGGAGAT
GCTCAAGAGAGGCTTACCCAGATTCTAAGTACTGTGAAAAACACATGCAC
AGAGGAAGAAACCGTGCTAGAAAATCTCTTGATCAGAATCAGACAACAAC
AACTCCTTTAACATCACCATCTCTCTCATTCACCAACAACAACAACCCAAG
TCCTACCTTGTCATCTTCTTCTTCATCTAATTCATCTTCTACTACTTATTCTG
CTTCATCTTCATCTATGGATGCTTACAGTAACAGTAATAGGTTTGGGCTTG
GTGGAAGTAGTAGTAACACTAGAGGTTATTTCAACAGCCATTCTCTTGATT
ATCCTTATCCTTCTACTTCACCTAAACAACAACAACAAACTCTTCATCATGC
TTCCGCTTTGTCACTTCATCAAAATACTAATTCTACTTCTCAGTTCAATGTC
TTAGCTTCTGCTACTGACCACAAAGACTTCAGGTACTTTCAAGGGATTGG
GGAGAGAGTTGGAGGAGTTGGGGAGAGAACGTTCTTTCCAGAAGCATCA
AGATCATTTCAAGATTCTCCATACCATCATCACCAACAACCGTTAGCAACA
GTGATGAATGATCCGTACCACCACTGTAGTACTGATCATAATAAGATTGATC
ATCATCACACATACTCTTCATCTTCATCATCTCAACATCTCCATCACGACCA
TGATCATAGACAGCAACAGTGTTTTGTTTTGGGTGCTGACATGTTCAACAA
ACCTACAAGAAGTGTCCTTGCAAACTCATCAAGACAAGATCAAAATCAAG
AAGAAGATGAGAAAGATTCATCAGAGTCTTCAAAGAAGTCTCTACATCACT
TCTTTGGTGAGGACTGGGCACAGAACAAGAACAGTTCAGATTCTTGGCTT
GACCTTTCTTCCCACTCAAGACTCGACACTGGTAGCTAAatatgaagatgaagat
gaaatatttggtgtgtcaaataaaaagcttgtgtgcttaagtttgtgtttttttcttggcttgttgtgttatgaatttgtgg
ctttttctaatattaaatgaatgtaagatctcattataatgaataaacaaatgtttctataatccattgtgaatgttttg
ttggatctcttctgcagcatataactactgtatgtgctatggtatggactatggaatatgattaaagataag

Supplemental table 2. Details of the mutation analysis result.

Target	Vector	Replicate	Transgenic lines	Mosaic	Heterozygous	Biallelic	Homozygous	No. mutants	Mutation rate (%)	Average (%)
LsPDS	pKSE401	1	70	4	0	1	2	7	10.00	15.24 ± 5.77
		2	63	5	1	1	2	9	14.29	
		3	56	9	1	2	0	12	21.43	
	pZKD672	1	60	11	2	2	3	18	30.00	38.59 ± 8.13
		2	53	9	3	4	2	18	33.96	
		3	39	13	3	3	2	21	53.85	
	pZKD673	1	67	22	1	11	9	43	64.18	69.38 ± 9.81
		2	49	18	0	7	6	31	63.27	
		3	57	23	2	13	8	46	80.70	
LsGPP2	pKSE401	1	21	6	1	1	0	8	38.10	28.74 ± 8.10
		2	29	7	0	0	0	7	24.14	
		3	25	6	0	0	0	6	24.00	
	pZKD672	1	88	16	3	4	0	23	26.14	41.85 ±15.07
		2	19	6	0	3	2	11	57.89	
		3	20	8	1	3	2	14	70.00	
	pZKD673	1	23	15	0	5	2	22	95.65	81.22 ± 12.92
		2	22	13	1	2	1	17	77.27	
		3	41	20	2	1	6	29	70.73	
LsBIN2	pKSE401	1	18	1	0	0	0	1	3.57	14.20 ± 9.01
		2	37	2	2	1	0	5	13.51	
		3	34	5	1	1	1	8	33.33	
	pZKD672	1	28	4	1	1	2	8	28.57	24.23 ± 3.89
		2	57	4	4	2	2	12	21.05	
		3	39	3	3	3	0	9	23.08	
	pZKD673	1	28	8	3	2	3	16	57.14	54.00 ± 5.63
		2	40	11	1	3	4	19	47.50	
		3	68	24	4	6	5	39	57.35	

Supplemental table 3. Summary of the mutation types by each vector.

Target	Vector	Mosaic	Heterozygous	Biallelic	Homozygous	Total
LsPDS	pKSE401	9.52	1.06	2.12	2.12	14.81
	pZKD672	21.71	5.26	5.92	4.61	37.50
	pZKD673	36.42	1.73	17.92	13.29	69.36
LsGPP2	pKSE401	25.33	1.33	1.33	0.00	28.00
	pZKD672	23.62	3.15	7.87	3.15	37.80
	pZKD673	55.81	3.49	9.30	10.47	79.07
LsBIN2	pKSE401	8.99	3.37	2.25	1.12	15.73
	pZKD672	8.87	6.45	4.84	3.23	23.39
	pZKD673	31.62	5.88	8.09	8.82	54.41

All the data indicates the percentages (%) of the total transgenic plants from all the 3 replicates.

Supplemental table 4. Details of the predicted off-target sites.

Target	Off-Target sites	Sequence (5‘-3’)	No. mismatch	CFD score	Chromosome	Position	Forward primer	Reverse primer
LsPDS	On-target	GGCCACCGAGTGACTCGATGTGG	0	1	Lsat_1_v8_lg_4	146413913	GGTGGACAGGCTTATGTTGAGG	ACGTATACCAGGGGTAGCGAAA
	OT1	GtCCACtagGTGACTCGATGAGG	4	0.34	Lsat_1_v8_lg_7	96669987	ATCTTGAAACTTTGTCAATTATCCT	CGTTATATTGGTTTTCAATGCTGG
	OT2	GGCtACCtAGTGACaCaATGTGG	4	0.27	Lsat_1_v8_lg_4	232502953	TTCTGGGTTGTCACACTTGG	TCAAAATGTGATAGCCCGCA
	OT3	GGCCACttAGTGtCaCGATGTGG	4	0.08	Lsat_1_v8_lg_7	40916203	ATCAACCTACACACCTCCCA	GTTTGCTGCATCAAGGCATT
	OT4	GGCCACttAGTGcCaCGATGTGG	4	0.06	Lsat_1_v8_lg_4	274911468	AGCCACATCATGGGAACGAAT	CCAGATGAGGGAGCACTAACC
	OT5	GGCCACCcAGgGcCTCGAgGGGG	4	0.01	Lsat_1_v8_lg_1	123288963	ATGTGCAGTTAGGTCACATACATC	GGGATTCCAAGTCTTTAACCTTAT
LsGPP2	On-target	ACGACAAGTTGCAGACATCACGG	0	1	Lsat_1_v8_lg_7	193637979	CTCACCCAACCGCTCATCTT	CCCCTCCTTCTGGTAGTTGG
	OT1	ACGAgAAGTTGaAGACATCAAGG	2	0.42	Lsat_1_v8_lg_5	8748135	AGTGTTCCTTGAAACCTTGTGA	ATAATCATAGGCATGGACTGGC
	OT2	ACGAgAAGTTGaAGACATCAAGG	2	0.42	Lsat_1_v8_lg_4	222241665	GTTTTAAGTCGTGAGTGGTATAG	CTTTCCTAGTCAACCTTAGTCCA
	OT3	AtGAgtAGTTGCAGACATCATGG	3	0.39	Lsat_1_v8_lg_6	48019106	ATATCAGCCACTGCTTTCACTT	TACACTCTCTTGATCGTGACCT
	OT4	ACcAaAAGTTGCAGACATgAAGG	3	0.02	Lsat_1_v8_lg_8	53550380	ATTTCTCTCTCTGCTTGATAGCG	GTTCCTTCTGAGCCACCGAAAT
	OT5	tCGAgAAGTTaaAGACATCAAGG	4	0.42	Lsat_1_v8_lg_2	124351644	CCAAATGGCGAAACCCACATGA	TCACTGAAATTGCGGGTACTTCT
LsBIN2	On-target	ATCACAGTGATGCTCGTCAAAGG	0	1	Lsat_1_v8_lg_1	164970342	TGTGGTAGGGGCTGGATCTT	GAGGCTTCAAGTCCCTGTGG
	OT1	ATCACAGTGcgGCTCGTCAAGGG	2	0.4	Lsat_1_v8_lg_7	176558556	GACCACAATCTTGTATTCCCCA	TCCTACTACTCGAAGTCCCAAA
	OT2	caCACAGTGATGtTCGTCAAGGG	3	0.5	Lsat_1_v8_lg_6	186005438	AGAATTCTGATGACCACTGTGA	GGCTCAGTTATTCCTCGATTCA
	OT3	taCACAaTGtTGCTCGTCAACGG	4	0.74	Lsat_1_v8_lg_6	117482620	TGAAGGTGTAAGTCCACAAGTC	TGCTTCGTCACTTCTGTTGAA
	OT4	caCACAGTGATGtTCaTCAAAGG	4	0.5	Lsat_1_v8_lg_6	91910280	ATGCTACACGCTTGTGTTATAGA	CATTCACGAGGTAGCGAAGATG
	OT5	ATaACAaTGAaGCTCGTtAATGG	4	0.41	Lsat_1_v8_lg_1	135456562	AGTCATAATTTAGACCTTAATGGCA	ATCGATTCCGAAAGGTCACAAAA

Supplemental table 5. The primers used in this study.

Primer	Sequence	Notes
PTG-F	cgaacgatactcgagtaaATCAGCTCCACCCCCGAAAA	Construction of pZKD672
PTG-R	tggtccttgtaatccatctagAATctgcaagaaataatcacca	
UBQ10p-F	taaaacgacggccagtgccGGTACCGATCAGGATATTCTTGTTTAAGA	Amplification of the Arabidopsis UBQ10 promoter
UBQ10p-R	CTGTTAATCAGAAAAACTCAGATTA	
HspT-F	atatgaagatgaagatgaaatattt	Amplification of the Arabidopsis Hsp terminator
HspT-R	tgttgacctgcaggcatgcaagcttcttatctttaatcatattcca	
GRF5-F	AGTTTTTCTGATTAACAGATGATGAGTCTAAGTGGAAGT	Amplification of the codon optimized Arabidopsis GRF5 gene
GRF5-R	tcatcttcatcttcatatTTAGCTACCAGTGTCGAGTCT	
qRT-LsACT-F	CTGGTGTGATGGTAGGTATGG	Primers for the internal control LsACT in quantitative real-time PCR
qRT-LsACT-R	CTCGTTGTAGAAAGTGTGATGC	
qRT-Cas9-F2	GCAGGAGATTGGCAAGGCTA	Primers for Cas9 in quantitative real-time PCR
qRT-Cas9-R2	GCCATTGGCCAGAGTGATCT	
qRT-LsPDS-F	GCCACCGAGTGACTCGATG	Primers for sgRNA in quantitative real-time PCR
qRT-LsPDS-R	CGACTCGGTGCCACTTTTTC	
LsPDS-U6-F	attgGCCACCGAGTGACTCGATG	Primers for LsPDS-pZKD671
LsPDS-U6-R	aaacCATCGAGTCACTCGGTGGC	
LsGGP2-U6-F	ATTGcgacaagttgcagacatca	Primers for LsGGP2-pZKD671
LsGGP2-U6-R	AAACcgacaagttgcagacatca	
LsBIN2-U6-F	ATTGtcacagtgatgctcgtcaa	Primers for LsBIN2-pZKD671
LsBIN2-U6-R	AAACttgacgagcatcactgtga	
LsPDS-PTG-F	TGCAGGCCACCGAGTGACTCGATG	Primers for LsPDS-pZKD672 and LsPDS-pZKD673
LsPDS-PTG-R	AAACCATCGAGTCACTCGGTGGCC	
LsGGP2-PTG-F	TGCAacgacaagttgcagacatca	Primers for LsGGP2-pZKD672 and LsGGP2-pZKD673
LsGGP2-PTG-R	AAACtgatgtctgcaacttgtcgt	
LsBIN2-PTG-F	TGCAatcacagtgatgctcgtcaa	Primers for LsBIN2-pZKD672 and LsBIN2-pZKD673
LsBIN2-PTG-R	AAACttgacgagcatcactgtgat	
LsBIN2-seq-F	TGTGGTAGGGGCTGGATCTT	Amplification of the LsBIN2 target region
LsBIN2-seq-R	GAGGCTTCAAGTCCCTGTGG	
LsGGP2-seq-F	CTCACCCAACCGCTCATCTT	Amplification of the LsGGP2 target region
LsGGP2-seq-R	CCCCTCCTTCTGGTAGTTGG	
LsPDS-SEQ2F	GGTGGACAGGCTTATGTTGAGG	Amplification of the LsPDS target region
LsPDS-SEQ2R	ACGTATACCAGGGGTAGCGAAA	
Cas9-check-F2	TCTACCTGTACTACCTCCAGAATGGC	Check the transgenic positive plants
Cas9-Check-R2	CCTCACATCGTAAACCTTGTAGTCCC	
